# Current status of induced pluripotent stem cells in cardiac tissue regeneration and engineering

**DOI:** 10.1186/2050-490X-1-6

**Published:** 2013-11-08

**Authors:** Zhiqiang Liu, Jin Zhou, Haibin Wang, Mengge Zhao, Changyong Wang

**Affiliations:** Department of Advanced Interdisciplinary Studies, Institute of Basic Medical Sciences and Tissue Engineering Research Center, Academy of Military Medical Sciences, 27 Taiping Rd, Beijing, 100850 P.R China; Department of Chemical Engineering and Materials Science, Michigan State University, East Lansing, MI 48824 USA

**Keywords:** Cell therapy, iPSCs, Myocardial infarction, Regenerative medicine, Tissue engineering

## Abstract

Myocardial infarction (MI) is associated with damage to the myocardium which results in a great loss of functional cardiomyocytes. As one of the most terminally differentiated organs, the endogenous regenerative potentials of adult hearts are extremely limited and insufficient to compensate for the myocardial loss occurring after MI. Consequentially, exogenous regenerative strategies, especially cell replacement therapy, have emerged and attracted increasing more attention in the field of cardiac tissue regeneration. A renewable source of seeding cells is therefore one of the most important subject in the field. Induced pluripotent stem cells (iPSCs), embryonic stem cell (ESC)-like cells that are derived from somatic cells by reprogramming, represent a promising candidate due to their high potentials for self-renewal, proliferation, differentiation and more importantly, they provide an invaluable method of deriving patient-specific pluripotent stem cells. Therefore, iPSC-based cardiac tissue regeneration and engineering has been extensively investigated in recent years. This review will discuss the achievements and current status in this field, including development of iPSC derivation, *in vitro* strategies for cardiac generation from iPSCs, cardiac application of iPSCs, challenges confronted at present as well as perspective in the future.

## Review

Myocardial infarction (MI), an ischemic heart disease that usually leads to great loss of cardiac tissue and even heart failure, is the leading cause of death through the world [1]. After MI, it is impossible for ischemic heart to restore the scarred myocardium because of its limited regenerative capacity [2]. For the late-stage patients with MI, the only choice is heart transplantation, which is constrained by the insufficiency of donor organs [3]. Recent studies suggested that regeneration or repair of ischemic cardiac tissue may be achieved by cell replacement, i.e. transplantation of cells, especially functional cardiomyocytes to repair or replace damaged myocardium.

Seeding cells used in cardiac regeneration must be of multipotency, at least, into cardiac lineage. In addition, a high capacity of self-renewal and proliferation is also required [4]. In the past decades, many cell types have been used for cardiac regeneration, including skeletal myoblast, primary cardiomyocytes, mesenchymal stem cells (MSCs) and embryonic stem cells (ESCs) [5–9]. In comparison, ESCs have many potential advantages due to the highly proliferating and cardiomyogenic potentials [10]. Various strategies are available to produce sufficient ESC-cardiomyocytes (ESC-CMs) for replacement therapy [11–14], however, the further application of ESCs in cardiac tissue engineering is still hampered due to ethical problem, immune response, *etc.*[10]. In 2006, a breakthrough has been made that ESC-like cells have been derived from somatic cells by ectopic expression of OCT3/4, Sox2, KLF, c-Myc (induced pluripotent stem cell, iPSC) [15]. iPSCs are highly similar to ESCs in biology. Under suitable conditions, iPSCs could long-term propagate in undifferentiated state or differentiate into many other cell types, including functional cardiomyocytes [16, 17]. Furthermore, the derivation of iPSCs avoid the destruction of embryos and enabled the possibility to obtain patient-specific pluripotent stem cells, providing a promising resolution for clinical immune responses in cell transplantation [18]. Therefore, the development of cell reprogramming or iPSC technology may open up a new perspective to the quickly progressing field of cell-based therapy.

### Establishment of iPS cells by cell reprogramming and their implication for regenerative medicine

#### ***Development of cell reprogramming***

The concept of induced pluripotency was not innovatory. Some other strategies to induce pluripotency have been long developed, such as somatic cell nuclear transfer(SCNT), fusion of somatic cells with ESCs [19].

The pioneering work of directly reprogramming somatic cells into ESC-like state was done by Takahashi and Yamanaka [15]. They applied 4 factors, Sox2, Oct4, and KLF4, c-Myc (termed Yamanaka’s factors later), out of the screened 24 candidate genes to induce pluripotency from mouse embryonic or adult fibroblast; the resulted cells demonstrated a high similarity in morphology, self-renewal and multipotency to ESCs. When performed blastocyst microinjection, these cells formed chimeric embryos. However, the study failed to obtain live chimeric mice with the iPSC line. Closely behind, another independent group in America also successfully derived mouse iPSCs with the same set of factors and further, they obtained live chimeric mice [20]. In the same year, human iPSCs were also successfully established [21].

The development of direct reprogramming was a milestone in stem cell research. It provided an invaluable seeding cell resource for regenerative medicine and tissue engineering. Therefore, since the first derivation of iPSCs was reported, the cells have attracted extensive attention throughout the world. Though most investigators initially followed to use Yamanaka’ factors, it was soon shown that not all of the 4 factors were collectively necessary. For example, NANOG and LIN28 were demonstrated to be able to replace c-MYC and KLF4, Sox1 and Sox3 can replace Sox2 [22]. Further, different group demonstrated that omission of one or more of the 4 factors in some conditions was still sufficient to reprogram somatic cells into iPSCs [23, 24].

Different from SCNT, direct reprogramming strategy (iPSC strategy) is more easy to apply across the species. In the former development, it has been difficult to establish NT-ESC lines in some species. For instance, efforts in establishing rat NT-ESCs were not successful constantly until decade years after the strategy development, while no human NT-ESC line has been established yet. However, the iPSC strategy was extended rapidly across species since the initial derivation of mouse iPSCs. In less than 5 years, iPSCs were successfully derived in many other species, including rhesus, pig, rat, canine, rabbit, Sheep; bovine (Table 1). The cell origins for iPSC derivation were also extended to a range of other cell types (besides fibroblasts), including pancreatic beta cells, lymphocytes, liver, stomach, beta cells, neural progenitor cells, keratinocytes, adipose stem cells, blood, hematopoietic cell, melanocytes, cord blood cells, dental tissues, circulating T cells, endothelial cells, renal tubular cells, *ect* (Table 1).

**Table 1:** **Development of cell reprograming**

Events		Time	Ref.
**Establishment of iPS cells**	mice	2006	[15]
	human	2007	[21]
	rhesus	2008	[25]
	Pig, Rat	2009	[26, 27]
	Canine; rabbit	2010	[28, 29]
	Sheep; bovine	2011	[30, 31]
**Cell origin for iPSCs production**	fibroblast ;	2006;	[32]
	skin; pancreatic beta cells; liver, stomach, beta cells, neural progenitor cells; keratinocytes	2008	[33–38]
	Adipose stem cells; blood; Hemotopiotic cell; Melanocytes; Cord blood cells;	2009	[39–43]
	dental tissues; circulating T cells;	2010	[44, 45]
	endothelial cells; renal tubular cells;	2011	[46, 47]
**Reprogramming strategies**	Retroviral;	2006;	[15]
	ectopic expression;	2007;	[20]
	ectopic expression; lentiviruses; onintegrating adenoviruses; Plamid; not integrate; free of exogenous genes.;	2008;	[33, 34, 37, 48–51]
	polycistronic; reprogramming proteins.	2009;	[52–56]
	piggyBac (PB) transposition; nonintegrating episomal vectors; recombinant proteins;		
	'minicircle’ DNA	2010;	[57]
	miRNA;	2011;	[58]

The initial development of direct cell reprogramming is based on integrating viral vectors which integrate randomly into the host genome. Risks exist for reactivation of the viral transgenes, such as c-Myc, an oncogene whose reactivation will result in tumor formation [15]. Moreover, integrated provirus may change the neighboring gene expression of receipts. In 2003, Hacein-Bey-Abina and his colleagues have already observed oncogenesis in SCID children who had received the transplantation of retroviral gene-modified haematopoietic stem cells [59]. Therefore, investigators put great efforts on exploring safer vectors to make iPSC more therapeutically applicable. In these years, many other reprogramming vectors and methods were appearing, mainly divided into (i) virus [33], (ii) DNA [57], (iii) RNA [58], and (iv) protein [52]. Some researchers established transgene excision system to generate iPSCs, such as the Cre/LoxP recombination system [60], the moth piggyback transposon system [53]. In these systems, the integrated extraneous genes would be excised after transduction of target cells. Zhou et al. successfully developed a DNA-free strategy to generate iPSCs in which recombinant proteins were used instead of the transcription factors [52]. Though the reprogramming efficiency is rather low (about 1000-fold lower than that of retroviral system), it’s definitely an important advance in that gene transfer is dispensed. In the most recently, RNA was also successfully applied to generate iPSCs [58].

#### ***IPSC implication for regenerative medicine***

The rapidly progressing field of iPSCs demonstrated vast implications in regenerative medicine and tissue engineering. The high similarity of iPSC to ESC make it a potential replacement of ESCs in therapeutic use, and many advantages make it superior to ESCs:1) the derivation of iPSCs bypass the ethical controversy surrounding ESCs whose derivation get involved in destruction of human embryos; 2) the direct reprogramming somatic cells into ESC-like state enable the possibility to obtain patient-specific stem cells of highly pluripotency, providing a promising perspective to minimizing the immune rejection in cell replacement therapy. Additionally, it needs not to perform invasive procedures to obtain candidate cell because of the extensity of cell types suitable for direct reprogramming; 3) to obtain autologous normal cells from patients of genetic diseases. Fanconi anaemia is a disease of severe genetic instability. Fibroblasts or keratinocytes derived from the patients carry severe genetic defects which do not allow patient-specific iPS cell generation. However, the genetic defect can be corrected with lentiviral vectors encoding FANCA or FANCD2 and corrected fibroblasts could be induced into iPS cells as efficiently as wild-type human fibroblasts. These iPS cells have equal capability as normal ones to differentiate into haematopoietic progenitors, whilst stably maintaining the disease-free phenotype *in vitro*[61]. The similar situation also exists in many other genetic hiPS cells. The derivation of autologous disease-free cells from these genetic-defect patient possess great therapeutic value when cell transplantation is needed.

To date, though developed for only several years, the great value of iPSCs in regenerative medicine and tissue engineering has been definitely displayed at least in the following aspects:

1. Be used as seeding cells for cell transplantation therapy. A typical instance of iPSC therapeutic application was Hanna J and his colleagues’ work. They demonstrated the feasibility to correct the defect by coupling gene targeting and direct reprogramming using a mouse model of humanized sickle cell anemia [62]. Similarly, Wernig and colleagues showed that iPS-cell-derived dopaminergic neurons could alleviate the disease phenotype in a rat model of Parkinson’s disease [63]. In addition, it has been confirmed too that iPSC treatment was effective for cardiovascular disease, spinal cord injury and many other diseases [64–67];
2. Be used for human disease models. Disease-specific iPSCs and their derivates would exhibit at least partial phenotype of the disease. One typical example is that iPSCs derivated from patients with heart disease differentiated into cardiomyocytes which would exhibit the specific disease behavior, such as long QT syndrome, Timothy syndrome, and LEAPORD syndrome [68–70]. Such disease-specific cells would be convenient and valuable in experimental research (such as pathogenesis, physiologic properties of these cardiomyocytes) because it is nearly impossible to obtain abundant samples from the patients. At present, several disease-specific iPSC line have already been established, including juvenile onset type 1 diabetes mellitus, Parkinson’s disease [71], amyitrophic lateral sclerosis [72], spinal muscular atrophy (SMA) [73];
3. Be used for high throughout drug screen. iPSCs could differentiated into many type of functional lineage-specific cells, which could be used for drug screening, drug effectiveness and toxicology tests instead of natural tissue. In the following part of the review, we will focus on the current status of iPSC’s application in cardiac tissue regeneration and engineering.

#### ***In vitro strategies for cardiac generation from iPS cells***

Of all stem cells potentially applicable in cardiac tissue engineering, ESC represents one of the most promising cell sources. ESCs possess potent capacities of long term expansion and efficiently cardiomyogenic differentiation, which allows for the creation of sufficient cardiomyocytes or supportive cardiac-lineage cells, such as vascular progenitor cells, smooth muscle cells and endothelial cells [1]. As ESC-like state cells, the advantages of ESCs in cardiac regeneration are manifested in iPSCs too.

The differentiation of iPSCs into cardiomyocytes *in vitro* was firstly reported in mouse iPSC lines by Christina and colleagues in 2008 [16]. The resulted cardiomyocytes showed typical features of ES cell–derived cardiomyocytes, including spontaneous rhythmical beating, expression of marker genes, expression of cardiomyocyte-typical proteins, spontaneous rhythmic intracellular Ca^2+^ fluctuations, presence of the β-adrenergic and muscarinic signaling cascade, and so on. But in the study, iPSCs showed a delayed and less efficient differentiation of beating EBs compared with ESCs. Almost at the same time, Genta *et al.* reported the direct and systematic differentiation of miPSCs into cardiac lineages [74]. MiPSCs were firstly induced into Flk1+ cells and sorted by FACS, then purified Flk1+ were systematically differentiated into endothelial cells, mural cells, arterial/venous/lymphatic endothelial cells and self-beating cardiomyocytes in different conditions, respectively. Different from Christina’s report, the differentiation properties of iPSCs observed in the study were almost the same to that of ES cells. Followed in 2009, Jianhua Zhang *et al.* firstly reported the cardiac differentiation of human iPSCs [17]. In the study, differentiation properities were also compared between hiPSCs and hESCs. The study showed that both hiPSCs and hESCs have a similar capacity for differentiation into nodal-, atrial-, and ventricular-like phenotypes as analysized by electrophysiology. HiPS and hES cell-derived cardiomyocytes share similar cardiac gene expression patterns, proliferation, sarcomeric organizations and they exhibited similar responsive pattern to β-adrenergic stimulation. However, the study observed a similar phenomenon as observed in miPSCs, that the differentiation efficiencies of hiPSCs varied with cell lines. These studies (both on mouse and human iPSC lines) coincidentally indicated that cardiomyogenic potentials of iPSCs could be line-specific.

In recent years, many other methods for differentiating iPSCs into cardiomyocytes have been developed (Table 2). Theoretically, any strategies and reagents differentiated ESCs into cardiac lineage may be applied to iPSCs due to their highly similarity. Actually, many methods used for iPSC cardiac differentiation are based on the previous studies in ESCs. BMP4 and activin A are potent factors that induced hESCs into cardiomyocytes [14]. Therefore, both of them are definitely candidate inducers to differentiate hiPSC into cardiomyocytes. In a direct differentiation system reported by Hideki Uosaki *et al.*, BMP4, activin A and bFGF were combined to induced iPSCs differentiation [75], the induced cardiomyocytes (cardiac troponin-T –positive) appeared robustly with 30–70% efficiency. In a EB-based differentiation sysytem reported by Paul W. Burridge, *et al*. [76], they combined BMP4, FGF2, polyvinyl alcohol, serum, and insulin to induced cardiac differentiation from hiPSCs and hESCs. At optimized concentrations, the differentiated rates of contracting EBs were enhanced up to more than 90%. Further, in the contracting hEBs, 64–89% of cells were cardiomyocytes. Many other inducers used in cardiac differentiation of ESCs were also confirmed effective to induced cardiac differentiation from iPSCs, including, 5-Azacytidine, ascorbic acid, cyclosporine-A and so on (Table 2). Of note, though the differentiation efficiencies varied among these methods, it is difficult to determine which one is absolutely superior without the direct comparison between different methods on the same cell line, because the differentiation of iPSCs toward cardiomyocytes may be cell-line-dependent as discussed above. Interestingly, in another report by Shinji Kaichi, *et al.*, investigators explored trichostatin A to improve iPSCs’ cardiac differentiation and found that treating iPSCs with trichostatin A seems to be useful to overcome cell line variation in the differentiation efficiency [77]. The reagent only or combined with other inducers may be promising in future application to facilitate cardiac differentiation of different iPSC lines, especially those could not be substituted, e.g., patient-specific iPSCs. Recently, Lee Carpenter *et al.* reported an efficient method for iPSCs differentiation into cardiac lineages [78]. In the study, iPSCs were differentiated as a monolayer. By combining PenStrep, monothioglycerol, ascorbic acid and several cytokines (including active A, BMP4 and bFGF), the method yielded more 50% cardiac-lineage cells (cardiomyocytes, endothelium or smooth muscle cells) out of total differentiated cells. Though the method is some complex and many inducers are involved, such a high efficiency for cardiac cell derivation from iPSCs would be clinically promising.

**Table 2:** **Cardiac differentiation from iPSCs**

Induce medium OR supplement	Species	Efficiency	Differentiation manner	Ref.
IMDM	Mice	~55% beating EBs	EB formation	[16]
α-MEM and coculture	Mice	Unclear	Direct differentiation	[74]
DMEM/F12	Human	~10% beating or bellow	EB formation	[17]
α-MEM	Human	Unclear	EB formation	[79]
DMEM	Human	Unclear	EB formation	[80]
5-AZ or BMP-4 or DMSO	Human	23.7% of cells by 5-AZ	EB formation	[81]
IMDM	Murine	Unclear	EB formation	[82]
Ascorbic acid	Mice; human	Unclear	Through Isl1(+) progenitors	[83]
IMDM	Mice	Unclear	EB formation	[84]
DMEM and coculture on END2 cells	Human	~12.8±3.5% beating EBs	Cell clumps	[85]
TSA	Mice	Unclear	Direct differentiation	[77]
FGF-10	Mice	Unclear	EB formation	[86]
RPMI+B27 (+Activin A/BMP4/bFGF)	Human	30-70% Beating EBs	Monolayer culture	[75]
Cyclosporin-A	Mice	Unclear	Through Flk1+ cells	[87]
DMEM	Mice	Unclear	EB formation	[88]
Insulin+ BMP4+FGF2	Human	>90%	EB formation	[76]
Sulfonyl-hydrazone-1	Mice	Unclear	EB formation	[89]
DMEM, EBM-2	Mice	~44.8%	EB formation	[90]
Stem Pro-34 medium + ascorbic acid + PenStrep + monothioglycerol + Activin A + BMP4 + bFGF	Human	More than 50%	Direct differentiation as a monolayer	[78]

### Current application of iPSC in cardiac tissue regeneration and engineering

The application of ESCs in cardiac tissue regeneration and engineering could be tracked back to 2002. Min JY *et al.* firstly injected ESCs in a MI rat model [91], and found that ESCs survived and regenerated cardiomyocytes in recipient myocardium and significantly improved heart function 6 weeks after cell transplantation. The study demonstrated the feasibility and validity of applying ESCs for myocardial repair and regeneration. Thereafter, the researches about ESC application in cardiac tissue regeneration and engineering grew rapidly. Many novel strategies emerged besides the direct transplantation of ESCs to optimize the therapeutic efficiency, such as injectable cardiac tissue engineering [9] (delivering ESCs in injectable biomaterials), pre-treatment of ESCs before transplantation [92], genetic modification [93], engineered cardiac tissue [94] and so on. Many ESC-derivated cells besides undifferentiated ESCs were explored to determine the optimal cell types, such as ESC-derived cardiomyocytes [11], ESC-derived endothelial cells [95], ESC-derived endothelial progenitors [96], ESC-derived pluripotent cells [97], and so on. Like the *in vitro* studies, the *in vivo* application of iPSCs in cardiac tissue regeneration and engineering also adopt a similar route as that of ESCs. However, as a newly emerging field, iPSC-based cardiac tissue regeneration and engineering is still preliminary.

The pioneer work that translates the concept of notion into practice was reported in 2009 by Timothy J. Nelson and his colleagues [64]. Within adult murine models of myocardial infarction, undifferentiated iPSCs were intramyocardial delivered by needle injection. Engrafted iPSCs restored myocardial performance, halted progression of pathologic remodeling in infarcted hearts and regenerated multi-lineage cardiac tissue, including cardiomyocytes (cardiac α-actinin positive), smooth muscle cells (Smooth muscle α-actin positive) and endothelial cells (CD31 positive). However, the observation in the study that iPSCs did not form detectable tumors in immunocompetent recipient hearts was soon disputed in subsequent reports [98, 99]. To minimize tumorigenic risk and optimize therapeutic efficiency of iPSCs for myocardial repair, Christina Mauritz and colleagues explored the usage of iPSC derived Flk+ cardiovascular progenitor cells in mouse model^56^. The data presented in the study demonstrated an encouraging efficiency of iPSC-derived cardiovascular progenitors for myocardial regeneration, but the tracking time in the study was rather short, only 2 weeks. Whether and how long the therapeutic benefits, as well as the survival of grafts could be maintained are unknown and deserve further investigation. Besides application in small animals, the therapeutic potential of human iPSCs for myocardial repair was also tested in higher animals (clinically more relevant animal models) [100]. Christian Templin *et al.* injected human iPSCs in pig models of MI and found that human iPSCs could be detected for up to 15w in pig myocardium. More importantly, they observed hiPSC-derived endothelial cells contributed to vascularization of infarcted myocardium.

Delivery method is a key factor that may influences the success of cell-based therapy.

From studies described above, though delivering iPSC with needle injection has acquired significant efficacy for myocardial repair, the limited cell number that could be delivered or detained in the target region is an apparent disadvantage for injection approach. Therefore, more effective method for delivering iPSCs was also developed.

Innovative from precious studies based on needle injection, Bo Dai and colleagues explore an tissue engineering strategy to create a tri-cell patch using iPSC-derived cardiomyocytes, endothelial cells and embryonic fibroblasts for myocardial repair in mice, resulting in significantly more cell survival, enhanced left ventricle (LV) function and reduced LV fibrosis. By present, this appears to be the most successful application of iPSCs in cardiac tissue engineering [101, 102].

Overall, though the *in vitro* strategies on the generation of cardiomyocytes from iPSCs are rapidly progressing, the *in vivo* application of iPSCs in cardiac tissue regeneration and engineering is still in infancy. Comparison between *in vitro* and *in vivo* studies may indicate that researchers are more cautious about *in vivo* application of iPSCs. To promote the future therapeutic application of iPSCs, more effective strategies for cell delivery need developing, novel means to enhance the survival, engraft and therapeutic efficiency of transplanted cells should be explored, the long-term validation and safety after iPSCs transplantation as well as the optimized cell population need to be determined.

### Current challenges confronted in iPSC-based cardiac tissue regeneration and engineering

Although many studies available supported that iPSCs could be a potential substitution of ESCs and represent a promising cell sources for cardiac tissue regeneration, lots of challenges remain and must be overcome prior their clinical applications.

1. Reprogramming efficiency is still low and reprogramming mechanisms are not exactly elicited. These years, though reprogramming technologies progressed at a high-speed pace and various strategies or augmented reagents appeared, the virus-mediated reprogramming still seems to be the most effective way. In addition, the derivation of iPSCs is not repetitive. The number of insertions of exogenous genes, the locus of gene insertion as well as the extent of reprogramming could vary among each reprogramming, even though the original cells were the same. One direct consequence in therapeutic use may be that one iPSC line is effective in myocardial regeneration and repair but another not. Actually, it has been founded that the pluripotency, including cardiomyogenic potential differs greatly among iPSC lines [77]. The resolution of the problems will largely depend on the better understanding of the reprogramming mechanisms.
2. Common to ESCs, the tumorigenicity is also an obstacle preventing the further application of iPSCs. Recently, two independent studies in mice and rats consistently indicated that intramyocardial transplantation of iPSCs is accompanied with a high tumorigenic risk [98, 99]. Furthermore, it seems that iPSCs derived progenies retain tumorigenic potential too [103]. Some investigators have tried to obviate the tumorigenic capacity of iPSCs reprogramming, such as excluding c-Myc in reprogramming [32]. Unfortunately, they do not work well in deed.
3. A practical strategy for iPSC differentiation and target cell purification is needed. The differentiation rates of iPSCs into cardiac-lineage cells varied among strategies and cell lines, but generally, not a single report is ideal to meet clinical standard. Additionally, an effective and practical method for target cell selection is desired as adverse cells could long term exist during iPSC differentiation and may result in unpredicted side effects after *in vivo* transplantation [103].
4. The immunogenicity of iPSCs should be considered renewedly. Given that iPS cells can be derived from patients themselves, it had been considered that iPSCs provided a possibility to overcome problems of immunological rejection associated with cell transplantation. However, a latest report by Zhao et el. showed that iPSCs may possess a higher immunogenicity than predicted and evoked immune response even in syngeneic recipient [104]. Some mechanisms for increased immunogenicity in iPSCs were proposed too by the study, such as abnormal overexpression of some genes. Accordingly, an effective strategy to control immunogenicity of iPSCs in reprogramming is indispensable on their way to clinical practice, and each iPSC line need to be strictly examined before transplantation, including patient-specific iPSCs.

In addition, there are also several fundamental challenges common to iPSCs and other cells in cardiac application, e.g., it has been reported that ESC-CMs possessed a low capacity for electrophysiological integration [105]; which could be an obstacle too for iPSC-based myocardial repair. Other challenges include poor retention and survival of transplanted cells in target regions, long-term efficacy, arrhythogenic risk, and so on.

### Perspective and conclusion

Just within six years, the experimental achievements in iPSC research have generated great expectations in both clinicians and patients. At present, the iPSCs have been beginning contributing to the treatment of cardiovascular and other diseases. On one hand, the disease models established from iPSCs have been confirmed effective and used for research of physiologic properties, pathogenesis of some diseases, including heart disease (e.g. Timothy syndrome), nervous system diseases(e.g. Parkinson’s disease) and many other genetic diseases. On the other hand, iPSCs have already been used in the drug screening for disease treatment [106, 107]. Despite these progresses, the therapeutic application of iPSCs for MI is still at an early stage (limited in animal models), no clinical application of iPSCs has come true. Thus, more effects are still needed to derive clinical-grade iPSC lines and overcome so many challenges.

## Conclusion

In summary, the iPSC research has been progressing with an amazing speed from the first onset, encouraging achievements have been unceasingly emerging and all these are just the start. Current problems referring to iPSC application in cardiac regeneration as well as in other areas of regenerative medicine should be looked with optimism. The scientific community is sparing no effort to push the advancements in iPSC basic research and its clinical use. Several novel techniques have also demonstrated promising perspectives related to the resolution of relative problems, such as novel chemicals/compounds or systems to enhanced reprogramming efficiency [108]; novel reprogramming notion to avoid tumorigenicity [109, 110]. Meanwhile, it should be objectively looked that each small step toward clinic would depend on the tremendous efforts in basic research. Despite the remarkable achievements in iPSC research within such a short time, it should be said that there is still a long way to go and many barriers to overcome before the true realization of iPSC application in clinical practice.

## Abbreviations

MI: Myocardial infarction
iPSC: Induced pluripotent stem cell
ESC: Embryonic stem cell
EB: Embryoid body
CM: Cardiomyocyte
FD: Familial dysautonomia
SCNT: Somatic cell nuclear transfer
SMA: Spinal muscular atrophy
FACS: Fluorescence-activated cell sorting
LV: Left ventricle
Vc: Vitamin C.
